# Developing guiding principles for technology-based rehabilitation program by engaging people with motor incomplete tetraplegia

**DOI:** 10.1186/s12984-022-01096-2

**Published:** 2022-11-24

**Authors:** Alison Bell, Namrata Grampurohit, Gabrielle Kains, Ralph J. Marino

**Affiliations:** 1 Jefferson College of Rehabilitation Sciences, Thomas Jefferson University, 901 Walnut Street, Suite 600, Philadelphia, PA 19107 USA; 2 Goldstar Rehabilitation, Narberth, PA USA; 3 Sidney Kimmel Medical College, Thomas Jefferson University, Philadelphia, PA USA

**Keywords:** Tetraplegia, Spinal cord injury, Neurologic rehabilitation, Qualitative research, Upper extremity, Technology

## Abstract

**Background:**

Technology-aided rehabilitation is well established in the field of neurologic rehabilitation. Despite the widespread availability, the development of technology-based interventions that incorporate perspectives of the people who will use them is lacking.

**Objectives:**

This qualitative study aims to understand how people with chronic motor incomplete cervical spinal cord injury view rehabilitation technology to improve upper extremity function and neuromuscular recovery to inform future intervention development.

**Methods:**

Seven participants with chronic upper extremity impairment due to spinal cord injury/dysfunction trialed five rehabilitation technology devices. After a 30–45 min trial for each device, participants engaged in a semi-structured interview. Interviews were analyzed using a qualitative approach to explore the experience using and understand features that support motivation to use of rehabilitation technology.

**Results:**

Qualitative analysis revealed three major themes: (1) devices must be flexible to meet diverse needs; (2) intervention protocols must be individualized to address unique needs and contexts of users; (3) intervention protocols should be developed and updated by a skilled clinician. These themes and subthemes were used to describe guiding principles to inform future intervention design.

**Conclusion:**

The experiences of people with cervical spinal cord injury can be elicited as part of the intervention design process to systematically develop protocols for future feasibility trials. The findings from this study can be used to inform the development of technology-aided rehabilitation programs to improve upper extremity function in people with chronic motor incomplete tetraplegia.

*Clinical trials registration number:* NCT04000256

## Background

Upper extremity paralysis is a consequence of cervical spinal cord injury (SCI) and a high treatment priority for individuals with tetraplegia [1, 2].

Best practice in the development of rehabilitation technologies and treatment programs includes listening to the voices of people with SCI [3, 4]. However, this step is often missing from research reports and is a barrier to successful clinical practice implementation of the intervention [5]. Engaging the people who will use the intervention early in the design process can allow researchers to understand the priorities and needs for the intervention and allow for the development of meaningful interventions that promote adherence [6].

The person-based approach to intervention design incorporates perspectives of the people who will use an intervention, attempting to understand their needs, priorities, and contexts [6]. The process guides researchers to hear the participants’ perspectives through formal qualitative inquiry as the first step in intervention design [6]. Our team has adopted the person-based approach to develop a technology-based upper extremity intervention program for home use for people with motor incomplete tetraplegia. Our team aims to design a protocol with low-cost equipment that users will be motivated to use in the home to support high levels of adherence to a rehabilitation program to improve upper extremity function. Adherence to rehabilitation programs describes the degree to which people follow the recommendations and schedules of health care providers [7]. This paper presents the findings of the qualitative inquiry. It offers guiding principles and key features of a home-based technology-aided activity rehabilitation program, focusing on the facilitators of home use based on the experiences of people with chronic motor incomplete tetraplegia.

## Methods

This research study uses a descriptive approach to understand the experiences of people with chronic motor incomplete tetraplegia using the technology. Semi-structured qualitative interviews were conducted with individuals with SCI over several visits as they used five different rehabilitation devices. Thomas Jefferson University’s Institutional Review Board approved the study. All participants provided informed consent prior to the start of the study. The protocol was registered on ClinicalTrials.gov (NCT04000256). The study was conducted between March 2019 and March 2020.

### Participants

Purposeful sampling to recruit participants with chronic motor incomplete tetraplegia was used. Incomplete motor injuries retain some motor function below the neurologic level of injury. Inclusion criteria was developed to recruit participants who would mirror the characteristics of participants in future studies using technology and who would be most likely to benefit from an activity based program to regain motor function.. People with motor incomplete spinal cord injuries demonstrate the greatest benefit from high intensity activity based programs compared to sensory incomplete and complete spinal cord injuries [8].

Subjects were recruited via flyers posted through the Regional Spinal Cord Injury Center of the Delaware Valley and included traumatic and nontraumatic injuries. Subjects were compensated $50 per visit for participation. Participants were screened using the following criteria.

Inclusion criteria:

- Cervical SCI, neurologic levels C1–C7
- Persevered motor function below the neurologic level of injury
- At least one arm with active shoulder flexion (reach with gravity eliminated) and at least one grasp pattern or able to move fingers
- At least 6 months post-injury
- At least one arm with greater than 50% normal passive range of motion in all upper limb joints, excluding the interphalangeal joints of the fingers
- Medically stable with no contraindications to the activities or to sitting
- 18 years of age or older and English speaking.

Exclusion criteria:

- Uncontrolled pain in the upper limbs
- Upper extremity amputations
- Unable to commit to at least three visits
- Surgical procedures (e.g., tendon transfers) or orthopedic trauma (e.g., fracture) within the past 3 months
- Mechanical ventilation
- Other neurological conditions.

### Procedure

Each participant completed up to 3–5 sessions (median = 3) in a research laboratory. The first session was general intake that included demographic information, interview of activities they were interested in working on and standardized measures of upper extremity function. Goal areas and standardized measures were used to inform study personnel for the purposes of setting up the equipment. In the subsequent sessions, they trialed devices under the direction and supervision of an occupational therapist and occupational therapy student. Device trials lasted about 30–45 min. After each device trial, the participants engaged in an audio-recorded semi-structured interview. The devices selected for trial were all commercially available, lower cost, and hypothesized to fit in a person’s home environment. These properties were chosen based on the goal of developing a lower cost home-based intervention.

Devices trialed included the SaeboRejoyce Rehabilitation System (Saebo, Charlotte, NC), Neofect Pegboard (Neofect, San Francisco, CA), Neofect Smart Glove (Neofect, San Francisco, CA), Therapy Mouse (LiteGait, Tempe, AZ), and Recovery Rapids (Games That Move You, Columbus, OH). The SaeboRejoyce Rehabilitation System is an arm workstation that interacts with games loaded onto a computer. The workstation includes gross motor and fine motor manipulation tasks that are unimanual and bimanual. The Neofect Pegboard is a digital pegboard that provides gamified training through visual and auditory feedback before and after peg placement. The pegs and digital pegboards include options for different pinch patterns, such as different shaped pegs and pegs of varying diameter. Cognitively complex patterns and shapes for the pegs can be selected. The Neofect Smart Glove includes a passive exoskeleton that extends over the forearm, wrist, and digits to measure movement with an accelerometer and bending sensor. The exoskeleton interacts with games loaded onto a computer. The Therapy Mouse is a small square-shaped wireless mouse that can be placed on different objects using Velcro to elicit the desired movement patterns for rehabilitation programs. The Therapy Mouse can interact with any computer as a standard mouse. The mouse was placed on objects such as a ball, cup, glass, stick, elastic band around the arm, and a bolster for bimanual practice. BigFish Online Games (Big Fish Games, Seattle, WA) were used with the mouse for this study. Recovery Rapids is a video game used with Microsoft Kinect (Microsoft Corp., Redmond, WA) and detects upper extremity movements for unimanual and bimanual raft simulation games.

### Semi-structured interview questions

The focus of inquiry was the features and factors that would support interest and ability to use rather than identifying the best device. Questions were structured to understand what would influence participants to use the technology in their homes as part of a rehabilitation program. Study investigator and another occupational therapy student interviewed participants. Field notes were taken during the interviews. The questions were based on the Technology Acceptance Model (TAM) [9] and developed by the research team with input from one individual with SCI (Table 1). TAM aims to describe how and when users will use a new technology based on perceived usefulness and perceived ease of use [9]. These factors influence attitudes and the intention to use the technology, affecting the technology’s ultimate acceptance or actual use [10]. The model is well established in health care [10] and aligns well with the person-based approach to intervention design with a shared focus on the eventual user of the technology or intervention. Questions were all followed with additional probes such as ‘why do you say that’ or ‘can you tell me more’ to explore the experience and allow participants to elaborate on their responses.

**Table 1:** Semi-structured interview questions

Focus	Question
Motivation	Do you find this system/training fun? Does this system/training capture your attention?
Engagement	Does this system/training keep you engaged throughout the session?
Challenge	Does this system/training offer a challenge for you? Is it too easy? What can make it difficult? Is it too hard? What can make it easy?
Limitation	Does the system/training target the limitations in movement you experience? Does the system/training miss any important training components?
Feasibility at home	Would you use this system/training at home? What challenges do you see with this system/training at home? What advantages do you see with this system/training at home? What about the time needed to do the training? How can you make time for this? What about space? Do you have space for this? What about interruptions in the home? What about internet or power connection for the system?
General probes	Tell me more Can you give an example? Can you explain this more?

### Research team and reflexivity

The research team includes authors and research assistants. NG has formal research training in qualitative methodology and intervention design. AB is a clinician with advanced practice knowledge and education in SCI rehabilitation. GK completed her clinical doctoral training in the research lab with NG. RM is a physician and researcher with extensive experience in SCI care and research. All authors and research assistants identify as female, except RM, who identifies as male. The interview and coding team had no prior interaction with study participants.

AB and NG completed the coding and thematic analysis. Regularly research meetings were held to review the data and reflect on impressions. Field notes and memos were used during the data collection and analysis process. Both researchers engaged in bracketing to address concerns that the researcher’s experience with SCI and rehabilitation technology may influence objectivity in the code and theme generation. Bracketing is a qualitative research method where researchers acknowledge and document any preconceptions [11]. Both NG and AB developed a diary entry of initial preconceived notions about the data and updated the diary during the data collection and analysis to acknowledge when their preconceptions may differ from the data. These reflections were discussed as part of the research meetings.

### Trustworthiness

Strategies to ensure the trustworthiness of the study were employed throughout the research process. During the data collection phase, triangulation was addressed by allowing each participant to trial multiple devices, the data was collected over a period of weeks for each participant, and field notes were taken by the interview teams. The data analysis stage included the use of two coders reviewing the data independently and together and regular research meetings to review reflections, observations, and memos from the coding process. An audit trail that includes the code book, resolved memos, notes on theme development, audio of interviews and transcripts of these interviews is available. Themes were confirmed via member checking with one participant, who endorsed all themes.

## Analysis

All interviews were audio-recorded and transcribed verbatim by a research assistant and then audited by a second assistant for accuracy. Total interview length for participants ranged from 41 to 64 min (mean = 51 min). The data were analyzed using a general inductive qualitative approach [12]. The general inductive approach to qualitative inquiry is guided by the research objective, in this case the desire to understand guiding principles for a technology based intervention for upper extremity motor recovery for home use. Guiding principles summarize essential features that are necessary to achieve intervention objectives, in this case, supporting adherence [6]. The findings arise from the raw data, however, are influenced by the research question. This approach follows a five-step process that includes cleaning the data, close reading of the text, code generation, code revision and finally refinement to major categories [12].

Transcripts were uploaded into Dedoose version 8.3.47b [13] and reviewed by the authors (AB and NG). Initial codes were developed in tandem. Code development was guided by the close reading of the transcripts and the research objective. A codebook was created and maintained with definitions, qualifications, and exclusions. After the initial codebook development, researchers coded transcripts individually and then reviewed for agreement between the coders (consensus). Regular research meetings occurred to discuss and resolve coding conflicts and add and revise codes or subcodes to the codebook. After 42% of the interviews were coded with consensus established, only one researcher coded the remaining transcripts. Research meetings continued to discuss additions or changes to the codebook. Data saturation was defined as no new codes emerged during the analysis. After coding four subject’s interviews, additions to the codebook were only subcodes and full saturation was seen after coding of six of seven subject interviews.

Final themes and the development of guiding principles were discussed and agreed upon by the research team.

## Results

Ten people contacted the research team to engage in the study. Two were unable to participate due to scheduling conflicts, and one did not meet inclusion criteria. Seven people met the inclusion criteria and participated in the study (Table 2).

**Table 2:** Participant demographics

	Age/Gender	Years since injury	Neurological level /AIS grade	GRASSP							
				Sensation Doral and Palmar Score out of 24		Strength Score out of 50		Prehension Ability Score out of 12		Prehension Performance Score out of 30	
				R	L	R	L	R	L	R	L
J01	58/F	1–5	C4/D	24	19	50	44	12	12	29	22
J02	26/M	10–15	C5/C	12	17	21	20	10	11	16	15
J03	42/F	10–15	C6/D	20	22	41	21	12	6	30	14
J04	40/F	16 +	C6/C	22	20	20	18	8	5	17	15
J05	57/F	1–5	C4/Non-traumatic injury	22	21	25	26	2	2	1	1
J06	30/M	1–5	C5/C	21	24	15	13	2	5	3	10
J07	37M	10–15	C8/C	23	22	35	40	10	12	21	25

AIS: American Spinal Injury Association Impairment Scale; GRASSP: The Graded Redefined Assessment of Strength, Sensation and Prehension; R: Right; L: Left

Three themes were identified (Table 3). Themse 1 and 2 contain subthemes; theme 3 does not.

**Table 3:** Themes and subthemes

Theme	Sub-theme	Sample codes	Sample quote
Devices Must be flexible to meet diverse needs	Multiple Treatment Target	Treatment targets	“The ones that incorporated more than a singular movement got more fun to me…but the ones that did not were pretty boring” J07
		Choice/variety	“the pegboard is extremely repetitive that you’re doing the exact same things. You’re just grasping a peg and putting it in a hole regardless of what the gameplay is” J07
	Just Right Challenge	Challenge	“it’s just hard for me to use…[I’d] probably give up after not working for a while” J06
	Gamification	Progress monitoring	“The reporting at the end is very interesting. Because then you can kind of see how you’re improving. I’m a competitive person so that makes it a little more interesting than just doing exercise” J01
		Game features	“I like the fact that you can play different games with it and it challenges your memorization skills” J05
		Gamification	“When you mess up, it’s like Oh, my God. Now let’s do this again. Let’s do this again. I know I can do it, I know I can do it. So it keeps you engaged as you want to do better. You don’t want the lower score on the system…..you want to be one of the high scores” J05
Intervention protocols must be individualized to address unique needs and contexts of users	Individualized goals	System match	“it was fun because I knew I was getting something out of it that was gonna benefit me” J06
		Movement demands	“Especially with my right arm reaching across, that was difficult… Reaching across there was difficult, but umm, uh, that’s what, that’s what I have to work on. It’s really weak.” J02
	Desire to use beyond therapy goals	Fits in my life	“I can also see me doing this with my granddaughter on my lap. And use doing it together” J05 pegs
	Desire to use independently	Assistance	“That’s the aspect I was looking at it for. Being able to move my arms on my own and at my own pace, without having to ask somebody” J05
Intervention protocols should be developed and updated by a skilled clinician	No subthemes identified	Fidelity	“One thing I did notice since it’s a lot of shoulders, especially in the beginning, I could hear my OT saying to me “Don’t chicken wing” and I felt myself starting to chicken wing” J01
		Need therapist training	“Maybe having a therapist run through it one time with somebody. Just so they’re using all the features. But I think there’s a real value in doing a circle back, you know after a couple weeks, after they’re using it. And I think that would hold true with any of the equipment’ J01

### Theme 1: Devices must be flexible to meet diverse needs

The diverse needs of users were most apparent when considering that no one device was universally described as positive or negative, suggesting that any device recommended for use must meet a wide range of needs, capabilities, and interests. The quotes from five participants describing the same device, the Neofect Pegboard, in very different ways support this theme.

“The pegboard is extremely repetitive that you’re doing the exact same things. You’re just grasping a peg and putting it in a hole regardless of what the gameplay is” J07

“I like the fact that you can play different games with it and it challenges your memorization skills” J05

“I think the pegboard would just become boring” J03

“… you’re not doing the same thing every day. I think that’s when it kind of gets boring, when you’re doing the exact same thing everyday. So this way you would have an ability to change it up” J01

“it was fun because I knew I was getting something out of it that was gonna benefit me” J06

Within this theme, four sub themes of ability to address multiple treatment targets, just right challenge, gamification and cognitive challenge were found.

### Multiple treatment targets

The intention to use the device and enjoyment was linked to the ability use it in different ways. Throughout the interview, respondents identified devices and games as positive when they challenged multiple treatment targets (such as shoulder, cardiovascular endurance, or hand function).

“I would spend a lot of time using this because just with the mouse you can target anything you want to target….I would do that a lot because …those muscles are really weak for me so.” J02

“The Rejoyce was definitely more fun. Just because their graphics and a whole variety of ways that you’re using it, you know, different…. The squeezing, the turning, pinching the key turning thing. There’s just a whole lot more to do with it” J03.

When the device was described as only addressing one treatment area, the intention to use was limited because it was perceived as less engaging.

“The ones that incorporated more than a singular movement got more fun to me…but the ones that did not were pretty boring” J07

### Just right challenge

The need for a just-right challenge was described by all participants. When the challenge was just above their current abilities, participants described the device positively.

“It totally kept me engaged because I had to actually challenge myself to move my hand to do the actual movements in the game. And sometimes my hand just didn’t want to work the way I wanted it too.” J05

A lack of fit between the difficulty and their current functional level resulted in a negative experience using the devices.

“It wasn’t really too hard to use. So I just went through it just to get it done; It wasn’t that fun. The games are easy to use” J06

“It’s just hard for me to use…[I’d] probably give up after not working for a while” J06

### Gamification

Gamification is the use of game experiences to engage or motivate the user. Participants consistently identified the competitive environment of scoring points or beating levels as motivators to continue training. Or improve performance from previous trials as positive features.

“When you mess up, it’s like ‘Oh, my God. Now let’s do this again. Let’s do this again. I know I can do it, I know I can do it.’ So it keeps you engaged as you want to do better. You don’t want the lower score on the system...you want to be one of the high scores” J05

“The reporting at the end is very interesting. Because then you can kind of see how you’re improving. I’m a competitive person so that makes it a little more interesting than just doing exercise” J01

The ability to play in the game environment was a positive aspect of the technology devices.

“It does challenge you, but it does it in a more playful manner that you don’t realize you’re doing it. Because you’re looking at the game and more so competing with the game and so it’s more like you’re doing it and don’t realize you’re doing it” J05

### Cognitive challenge

Participants largely enjoyed the addition of a cognitive challenge, even if they did not identify cognition as a goal. Cognitive challenges were available in puzzle games or as part of sequence and timing of game interaction.

“I like the ones that are cognitive along with it because then I don’t even realize I’m moving my wrist because I’m working on the challenge” J01

### Theme 2: Intervention protocols must be individualized to address unique needs and contexts of the users

Participants had unique rehabilitation priorities and varied contexts that included families and the availability of care partners. Users described the influence of contextual factors such as engaging family and caregiver assistance as important reasons why they would choose whether to use a device. Within this theme, sub themes of individualized goal areas, desire to use independently and desire to use device for more than just therapy were seen.

### Individualized goal areas

The alignment between the device’s ability to target their treatment priorities was a major factor in willingness to use at home. Participants all spoke of their individual rehabilitation needs and identified when a system would be able to target their treatment needs. The ability to target their needs influenced their intent to use the device. There was a large range in treatment needs that ranged from goals like strengthen or stretching to general fine motor goals to activity-based goals like writing. Some spoke explicitly about needing to tailor any program to their own specific therapy goals.

“I thought it did give me the chance to kind of work on the fine motor skills so I think that was helpful and it would probably be good for, for me when it comes to things like writing legibly and typing” J04

One participant identified that he didn’t have any goal areas he would work on with the device and despite having positive experiences using, clearly identified that he wouldn’t use it.

“[I would use] if I were like fresh out of inpatient and still working on recovery and hadn’t really plateaued at what my ability is. But for me personally, since I’m kind of like at the top of what my ability is capable of, I probably would not” J07

### Desire to use independently

Participants identified the ability to set up and use on their own as an essential factor. Devices that users could set up and use independently were devices that users reported they would use at home.

“That’s the aspect I was looking at it for. Being able to move my arms on my own and at my own pace, without having to ask somebody” J05

“I’d use this, this one at home. Yeah. Just because it is smaller. It’s easier to set up. I could do it myself ” J04

In contrast, devices with more complex set up were unlikely to be used, even if they were engaging.

“I’m afraid that someone who is not a professional would not be able to put [the device] on…and when you think about the expense, I don’t know how feasible that kind of technology is even though it’s fun” J04

Four out of seven participants discussed using the device in alternate ways to fulfill other roles like student, worker, parent, or grandparent. Engaging younger family members in the gaming was a frequent comment,

“I can also see me doing this with my granddaughter on my lap. And us doing it together” J05

“I think my son would definitely be interested in trying and maybe we could challenge each other” J03

One device has clear uses beyond therapy gaming. Participants expressed the desire to use the device for more than just a therapy program and include as part of their daily activities.

“I could use [it] for everything. I could use it both functionally and for fun. I could do games and work” J03

### Theme 3: Intervention protocols should be developed and updated by a skilled clinician

This category has no subthemes.

Only 1 participant said a device could replace therapy. The remaining participants wanted therapists to develop and update the intervention program they used at home.

“Maybe having a therapist run through it one time with somebody. Just so they’re using all the features. But I think there’s a real value in doing a circle back, you know after a couple weeks, after they’re using it. And I think that would hold true with any of the equipment’ J01

Six out of seven participants were concerned about maintaining fidelity in their treatment program. Participants could clearly describe ways to compensate and ‘cheat’ the rehabilitation program and expressed value in having therapists develop and update a treatment program.

“One thing I did notice since it’s a lot of shoulders, especially in the beginning, I could hear my OT saying to me “Don’t chicken wing” and I felt myself starting to chicken wing” J01

“With the mouse it’s really up to you and your therapists or whatever to make sure you’re targeting exactly what you want to target J02

### Developing guiding principles

Using the themes and subthemes, guiding principles for a technology-based upper extremity intervention program for home use were developed. In the person-based approach, guiding principles consist of two parts. The intervention design objectives describe what the intervention will address and key features that describe how those objectives are achieved. The intervention design objectives align with the themes of the qualitative inquiry and subthemes inform the key features. The goal of the qualitative inquiry was to summarize the features of the intervention to optimize the acceptability of the intervention and describe the key ingredients (Table 4).

**Table 4:** Guiding principles

Intervention design objectives	Key features
Identify devices that meet diverse needs	Device has capacity for multiple treatment targets (e.g., shoulder, elbow, wrist, hand) System offers multiple levels of challenge Game based strategies included in intervention Cognitive challenges included in gaming options
Intervention protocols must be individualized to address unique needs and contexts of the users	Goal setting is part of intervention design process Users should be independent with set up and use of equipment Opportunities for use with family members should be considered
Engage skilled clinicians to develop, monitor and update treatment programs	Individualized plan should be developed by skilled therapist Training may include adaptive equipment to facilitate independence with use of equipment Programs should be monitored to ensure correct application (avoid compensations) Programs should be routinely updated

## Discussion

This study identified the characteristics of upper extremity technology-based interventions that are important for people with chronic incomplete motor incomplete tetraplegia. Using the person based approach to intervention design as the first step in the intervention design process allows the priorities and perspectives of the person with tetraplegia to guide the development process. The guiding principles, developed through careful analysis of the data, provide a framework to build upper extremity technology-based interventions that are feasible in the home environment and can support adherence to the rehabilitation program.

Understanding key features of technology aided programs is of particular interest as technology availability continues to evolve. The changing landscape requires researchers and clinicians to routinely evaluate a device's features to determine if it is a good match. Understanding the consumer's perspective can inform technology adoption decisions in a rapidly evolving marketplace. The Nintendo Wii (Nintendo, Kyoto, Japan) in an example of the rapidly changing landscape of technology. It is a well studied as a technology aid to rehabilitation; however, the product is no longer manufactured or supported by Nintendo. Additionally, in a recent review of home-based interventions for neurologic UE, only 65% of studied devices were commercially available [14]. These guiding principles can be used to evaluate technologies that may be useful for people with chronic motor incomplete tetraplegia as they become available.

A person-based approach is vital to ensure the best use of limited resources. As described by the participants, resources include time in their habits and routines, family member supports, and finances. While cost is a well-described barrier to technology use for people with SCI [15], our subjects did not identify cost as a barrier (Two participants did identify one device they thought would be expensive but did not identify this as a barrier). This may be due to the efforts on the researchers to find lower cost devices for trial or that cost was not specified for the participants. The influence of other resources, most often family members for support, were frequently identified. However, our participants reported care partners would be able to help, even if they preferred to do it independently.

The results of this study are consistent with the work of other researchers. Standen [16] explored the facilitators and barriers to home-based use of a low-cost virtual reality system for people with stroke. This work identified the need for assistance as a barrier for adherence and use of this device in the home and is consistent with our study that identified the ability to set up and use independently as a critical consideration in the desire to use at home. Moineau [17] introduced a functional electrical stimulation device to clinicians and people with SCI and stroke, though, participants were not able to trial. Their qualitative exploration results were similar in that end-users described the interaction between their own physical and mental characteristics that would influence technology benefit and their willingness to use. Additionally, the study participants also expressed the need for training and follow-up with a clinician.

Of interest, study subjects consistently and accurately described positive features of devices as those that align with motor learning principles such as need for high repetition, feedback, individualized plans that were engaging [18]. Participants consistently identified the need for high repetitions, a fun game that engaged them in the experience, need for feedback on performance, and interaction that could be customized to their needs and simulate the tasks that were their personal goals. This alignment in key features between users' desires and best practice is encouraging for developing a successful home-based intervention that is feasible, acceptable, and effective for people with chronic motor incomplete tetraplegia.

All participants had internet access, and this did not present as a barrier. This is consistent with other work showing people with spinal cord injury regularly access the internet [19, 20] and embedding rehabilitation into this established context would not create additional hardships.

## Limitations

This study was limited by a small sample limited to a single geographic area. While data was obtained through multiple interviews with different devices, the only type of data was interview transcriptions. Future work in this area could be enhanced by employing additional strategies to ensure dependability, including returning transcripts to participants for review and enhanced member checking.

The intent of this work is to develop recommendations for a home-based intervention, however, the device trials occurred in a research lab. The mismatch between these contexts is another limitation of this work.

## Conclusion

The engagement of individuals with SCI through a systematic qualitative process helped describe the guiding principles that will inform the development of a future home-based upper extremity technology-aided intervention for people with chronic motor incomplete tetraplegia. The features that will support adherence are described and future intervention design trials can be informed by the perspectives of those who will use the intervention.

## Data Availability

Audio recordings and interview transcripts are not available for review to protect the participants identities.

## Abbreviations

SCI: Spinal cord injury
TAM: Technology Acceptance Model
AIS: American Spinal Injury Association Impairment Scale
GRASSP: The Graded Redefined Assessment of Strength, Sensation and Prehension
R: Right
L: Left
